# Internet-Based Interactive Health Intervention for the Promotion of Sensible Drinking: Patterns of Use and Potential Impact on Members of the General Public

**DOI:** 10.2196/jmir.9.2.e10

**Published:** 2007-05-08

**Authors:** Stuart Linke, Elizabeth Murray, Ceri Butler, Paul Wallace

**Affiliations:** ^2^Primary Care and Population SciencesUniversity College LondonLondonUK; ^1^Islington Mental Health Psychology ServicesCamden and Islington Mental Health and Social Care TrustLondonUK

**Keywords:** Alcohol, drinking, health promotion, Internet

## Abstract

**Background:**

Heavy drinking is responsible for major health and social problems. Brief interventions have been shown to be effective, but there have been difficulties in reaching those who might benefit from them. Pilot studies have indicated that a Web-based intervention is likely to be acceptable to heavy drinkers and may produce some health benefits. However, there are few data on how many people might use such a program, the patterns of use, and potential benefits.

**Objectives:**

The aim was to examine the demographic characteristics of users of a free, Web-based, 6-week intervention for heavy drinkers and to describe the methods by which users identified the site, the pattern of site use and attrition, the characteristics associated with completing the program, and the self-reported impact on alcohol-related outcomes.

**Methods:**

Cohort study. Visitors to the Web site were offered screening with the Fast Alcohol Screening Test, and those scoring above the cutoff for risky drinking were invited to register with the program. Demographic information was collected routinely at registration, and questionnaires were completed at the end of weeks 1 and 6. The outcome measures assessed dependency (Short Alcohol Dependency Data Questionnaire), harms (modified Alcohol Problems Questionnaire), and mental health (Clinical Outcomes in Routine Evaluation–Outcome Measure).

**Results:**

The records of 10000 users were analyzed. The mean age was 37.4 years, 51.1% were female, 37.5% were single, and 42.4% lived with children. The majority were White British, lived in the United Kingdom, and reported occupations from the higher socioeconomic strata. Over 70% connected to the Down Your Drink (Down Your Drink) site from another Internet-based resource, whereas only 5.8% heard about the site from a health or other professional. Much of the Web site use (40%) was outside normal working hours. Attrition from the program was high, with only 16.5% of registrants completing the whole 6 weeks. For those who completed the program, and the final outcome measures, measures of dependency, alcohol-related problems, and mental health symptoms were all reduced at week 6.

**Conclusions:**

The Web-based intervention was highly used, and those who stayed with the program showed significant reductions in self-reported indicators of dependency, alcohol-related problems, and mental health symptoms; however, this association cannot be assumed to be causal. Programs of this type may have the potential to reach large numbers of heavy drinkers who might not otherwise seek help. There are significant methodological challenges and further research is needed to fully evaluate such interventions.

## Introduction

Excess alcohol consumption, and the harm caused by it, is a major public health concern throughout the developed world [1-3]. Regular heavy alcohol consumption and binge drinking are associated with physical problems, mental health problems, antisocial behavior, violence, accidents, suicide, injuries, road traffic accidents, unsafe sexual behavior, underperformance at school, and crime. In the United Kingdom, alcohol misuse accounts for over 30000 hospital admissions for alcohol dependence, up to 70% of all admissions to accident and emergency departments at peak times, and up to 22000 premature deaths. The total cost of alcohol misuse to the health service was calculated to be £1.7 billion per annum, which though substantial, is much less than the total annual cost of alcohol-related crime (£7.4 billion) and lost productivity (£6.4 billion) [3]. Similar costs are identified in the United States, with the overall economic cost of alcohol abuse estimated at $184.6 billion, most of which was attributed to lost productivity [1]. Alcohol dependence is not confined to adulthood: in 2000, nearly 14% of 16- to 19-year-olds in Great Britain were found to experience dependence on alcohol [3].

Brief interventions seek to change views of the personal acceptability of excessive drinking and to encourage self-directed behavior change. They can be delivered by practitioners or as self-help materials. There is a substantial body of evidence demonstrating that brief interventions for individuals at risk can have significant impact on reducing alcohol consumption and, in some cases, alcohol-related harm when delivered both in primary and secondary health care settings [4-8]. However, their impact on public health has been severely limited, due partly to the reluctance of at-risk individuals to seek help [9] and partly to a lack of health care resources and the unwillingness of health care professionals to undertake these interventions [10]. General practitioners, for example, who are in a key position to deliver such interventions, rarely do so because of both lack of skills and fear of potential adverse effects on relationships with their patients [10].

Until recently, self-help materials were almost exclusively paper-based. However, the Internet has triggered a growth in more interactive self-help materials. It is thought that this interactivity is likely to enhance the potential for behavior change [11]. In public health terms, the Internet has the considerable advantage that once the Web site has been developed, the marginal cost of delivering the intervention to unlimited numbers of people is minimal, in marked contrast to face-to-face interventions. Approximately 60% of individuals in the United Kingdom report using the Internet regularly [12], and a recent telephone survey in Canada showed that current drinkers are more likely to have access to the Internet than abstainers [13]. Heavy drinkers have been shown to benefit from a PC Windows-based behavioral treatment program [14,15]. Younger people, who are most at risk of binge drinking [3], use new information and communication technologies such as the Internet and mobile phones in preference to more traditional sources of health information or health promotion [16]. Pilot studies have shown that people with drinking problems are willing to use screening tools on the Internet [17,18]. One study asked Web site visitors to complete a questionnaire about their alcohol use and provided a brief intervention in the form of feedback and advice [19]. A recent review identified a number of Internet-based screening and assessment tools (but few treatment or intervention-based applications), which were mostly directed at college students and usually required users to attend special sessions in an office [20-22].

One exception is the Alcohol Help Centre, which provided online personal feedback to users of an online eHealth service [23]. A modified version of this Internet-based tool was piloted in Finland. In a relatively small cohort study (n = 343), at 3-month follow-up, users had reduced their drinking compared to baseline [24].

Down Your Drink is a well-established, comprehensive, freely available, interactive Web-based treatment program for people with alcohol problems. An initial pilot study demonstrated the feasibility of the approach [25], and this present study seeks to extend what is known about the “natural history” of the site over a longer period and to describe the users and their outcomes. All advertising and promotion of the site ceased after the pilot study, but the Web-based program continued to be hosted on the Web site of Alcohol Concern, the United Kingdom’s premier charity addressing issues around alcohol.

The aims of the present study were to describe the patterns of use and self-reported effectiveness among users of Down Your Drink. The study set out to describe the demographic characteristics of users, the methods employed to identify and access Down Your Drink, the patterns of use, the demographic and clinical characteristics associated with completing the 6-week program, and self-reported changes in alcohol-related outcomes associated with use of Down Your Drink.

## Methods

This was a pragmatic cohort study of the first 10000 people who registered to use the site, after the end of the pilot phase (ie, after September 2003).

The study was approved by the Camden and Islington Local Research Ethics Committee.

Down Your Drink was developed with support from the Alcohol Education and Research Council as a Web-based interactive program of brief interventions to reduce alcohol consumption in heavy drinkers and is hosted on a single dedicated Web site [25].

**Figure 1:**
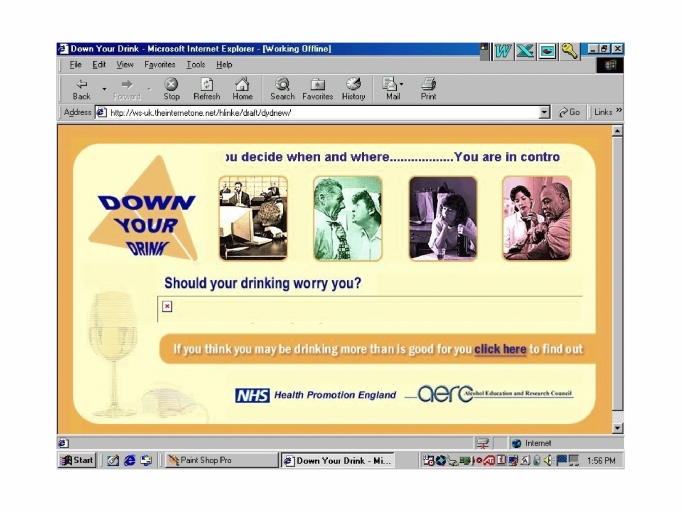
Down Your Drink home page

The animated home page invited visitors to assess their level of drinking by taking the Fast Alcohol Screening Test (FAST) [26]. Feedback was presented numerically and visually. The home page (Figure 1) also contained links to frequently asked questions (FAQs) about heavy drinking, information about the 6-week program, its authors, the research study, and the organizations sponsoring the site (The National Health Service, Alcohol Concern, Alcohol Education and Research Council, and the Royal Free and University College London Medical School ).

**Figure 2:**
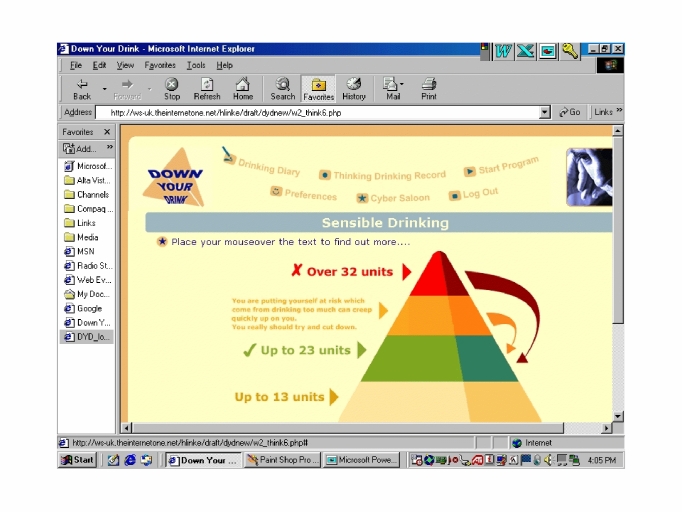
Sample Down Your Drink page

The Web site content was structured as a set of six consecutive intervention modules designed to be accessed by the registrants at weekly intervals. Once a given module had been completed, access to the subsequent module was barred for 7 days. The 6-week program was based on the stages of change model [27] and contained components common in brief interventions, including motivational enhancement [28], cognitive behavioral therapy [29], and relapse prevention [30]. Experience with the original print version of the program suggested that structuring the material over 6 weeks allowed users sufficient time to complete the various items and plan their behavior change. There were also components unique to a computer-based system, designed to exploit the interactivity and flexibility of the Internet (Figure 2 and Multimedia Appendix 1). These included an automated drinking diary and consumption calculator, online quizzes, interactive behavioral analysis of drinking situations (the “thinking drinking” log), blood alcohol concentration calculator, and intelligent email that sent reminders and controlled-drinking tips to an individual email address or as a Short Message Service (SMS) text message to a cell phone. Associated with the Down Your Drink program was a nonmoderated listserve for Down Your Drink users hosted by Yahoo groups where users of the program could exchange personal messages about their experience with the program and obtain peer support for their efforts in reducing their drinking.

### Recruitment

An off-line advertising campaign was run in September 2001 to coincide with the launch of the Web site and the early part of the pilot study. All off-line advertising ceased at the end of 2001 (Multimedia Appendix 2). The site was also registered with the Yahoo search engine and was listed in the information pages of a number of UK-based health-related periodicals. By the time this study started (September 2003), there had been no off-line advertising for this Web site for over 18 months. All visitors to the Down Your Drink site were invited to complete an initial assessment using the FAST [26]. The maximum score for the FAST is 16, and the cutoff point for risky drinking is 3. Those who scored 3 or above were advised that they were at risk from their drinking behavior and were directed to the free 6-week program. Those with lower FAST scores were also able to use the program. Visitors who wished to participate in the 6-week program were required to complete an online consent form (Multimedia Appendix 3) and provide registration data prior to accessing the site. Following this, they could enter the first week of the program.

### Baseline and Outcome Measures

In order to participate, users were invited to read the policy on confidentiality, complete the consent procedure, and then choose a username and password. Those who agreed to register were required to submit information about their age, gender, marital status, family composition, ethnicity, occupation, country of residence, and how they had learned about the site.

The primary outcome measure was the 14-item Short Alcohol Dependency Questionnaire (SADD), which measures dependency on alcohol [31]. This was selected in preference to a measure of alcohol consumption as, at that time, there was no direct measure of alcohol use validated for use on the Internet. Secondary outcome measures included an abbreviated (35-item) version of the Alcohol Problems Questionnaire (APQ), which assesses harm associated with heavy drinking [32], and the Clinical Outcomes in Routine Evaluation-Outcome Measure (CORE-OM), a 34-item measure of mental health symptoms [33]. For all these questionnaires, higher scores indicate greater levels of harm. All measures were completed online at the end of weeks 1 and 6. Respondents were able to review and check their responses by the using the back button on their browser. Respondents were not required to complete all the items in each questionnaire, thus allowing the possibility of differential response rates on each questionnaire.

All the data for the study were collected automatically on a live database located on a secure dedicated Web site. Access to this database was by password only and was restricted to members of the research team. This database was initially launched simultaneously with the Down Your Drink Web site in October 2001, but was withdrawn at the end of the pilot phase for re-development. The upgraded database was subsequently re-launched in September 2003, and the data presented here are from the first 10000 users after the launch of the revised database.

Usage data were collected automatically on the Down Your Drink Web server and analyzed by a Web server log file analysis program (Webalizer, version 2.01). These data were stored separately from the Down Your Drink registrant database. The usage data reported here are from a subset of the total participants in the study collected between January and December 2004.

### Analysis

Data for the first 10000 users were extracted from the live database on March 1, 2006, and transferred into SPSS, version 12.0.1 for Windows via Microsoft Access, where all data related to individual Web site users were linked using the unique identifier created by the Down Your Drink site.

These data were then subjected to frequency analysis and paired *t* tests were performed to compare the mean scores of Web site users at the beginning and end of the 6-week program. In addition, the demographic variables of age, gender, marital status (with or without partner), and family status (with or without children) were tested using a chi-square test to determine if a statistically significant relationship existed between particular demographic characteristics and the likelihood of completing the online program.

## Results

It took just over 27 months, from the launch date on September 24, 2003, to January 3, 2006, to complete recruitment of 10000 users.

### Demographic Characteristics of Down Your Drink Users

The self-reported demographic characteristics of the sample are shown in Table 1. It can be seen that approximately equal numbers of men and women used the program; a little over a third were single, and over 40% lived with children. Most users (81.9%) were White British, and over a quarter reported managerial and professional occupations.

The great majority (83.9%) lived in the United Kingdom, and 9.3% reported living in other English-speaking countries (United States, Canada, Australia, and New Zealand). Over 100 countries of residence were given by the remaining 6.7% of users.

**Table 1:** Demographic characteristics of Down Your Drink users

**Demographic Characteristic**		
	**Mean**	**SD**
**Age**	37.44	9.84
		
**Gender**	**No.**	**%**
Male	4891	48.9
Female	5109	51.1
		
**Marital Status**		
Single	3754	37.5
Married or living with partner	6246	62.5
		
**Living With Children**		
Yes	4244	42.4
No	5756	57.6
		
**Occupation**		
Managerial/professional	2579	25.8
Self-employed	862	8.6
Administrative/secretarial	854	8.5
Information technology	770	7.7
Academic	461	4.6
Housewife/househusband	431	4.3
Unemployed	353	3.5
All other	3690	36.9
		
**Ethnicity**		
White British	8185	81.9
White other	911	9.1
White Irish	527	5.3
Asian	135	1.4
Mixed	93	0.9
Black	69	0.7
Other	81	0.8
		
**Country of Origin**		
United Kingdom	8385	83.9
United States	554	5.5
Ireland	172	1.7
Australia	111	1.1
Canada	104	1.0
Other	674	6.7

### How Users Found Down Your Drink

Nearly three quarters of registrants (n = 7167, 71.7%) visited the Down Your Drink site from another Internet-based resource. Most of these connected via a link from another Web site (n = 4156, 41.6%) or from a search engine (n = 2900, 29.0%), whereas a smaller group responded to a banner advert on another site (n = 111, 1.0%). Relatively few registrants had been directed to Down Your Drink from the health service (n = 583, 5.8%).

### When Users Accessed Down Your Drink

**Figure 3:**
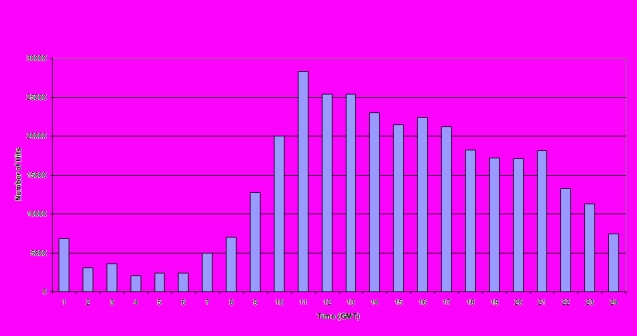
Mean hourly usage of Down Your Drink (Jan to Dec 2004)

The daily pattern of Down Your Drink use between January and December 2004 (represented by number of “hits” per hour) is shown in Figure 3. About 61% of the hits occurred between 9:00 am and 5:59 pm (Greenwich mean time [GMT]), with about 39% occurring between 6:00 pm and 8:59 am.

### Use of Down Your Drink

There was a high attrition rate from the program with only 1654 (16.5%) of the original 10000 registrants completing the 6-week program (Table 2). The greatest attrition occurred between the first and the second weeks, with much lower rates occurring thereafter.

**Table 2:** Number of users completing each week of the program. Percentages are the proportion of those registered, for example, 16.5% of all users that registered with Down Your Drink had completed the 6-week program by the time of the data extraction (March 1, 2006).

	**All Users**		**Male Users**		**Female Users**	
	**No.**	**%**	**No.**	**%**	**No.**	**%**
Registered	10000	100.0	4891	100	5109	100
Completed week 1	8933	89.3	4302	88.0	4631	90.6
Completed week 2	4020	40.2	1916	39.2	2104	41.2
Completed week 3	3006	30.1	1403	28.7	1603	31.4
Completed week 4	2411	24.1	1128	23.1	1283	25.1
Completed week 5	1928	19.3	887	18.1	1041	20.4
Completed week 6	1654	16.5	770	15.7	884	17.3

### Outcomes

In order to determine whether there were demographic or clinical characteristics that were associated with completion of the 6-week program, we compared the characteristics of those who completed the sixth week with those who had only completed the first week. We chose first week completers as the reference, as we did not have data on the outcome measures from those who had dropped out before completing the first week. We defined anyone who had done some part of week 6 as a completer, whether or not they filled in the questionnaires at the end of week 6. It can be seen that female users, users who were married or living with a partner, and users without children were more likely to complete the program than men, single users, or users with children (Table 3). The baseline responses of those users who completed the program indicated that they were less at risk of alcohol dependency and of harm from alcohol use at the time of entry into the program than those who subsequently dropped out (Table 3), although they were still at considerable risk of harm as judged by their baseline scores.

**Table 3:** Comparison of baseline demographic and clinical characteristics of those who completed the six week program (completers) with those who only completed the first week of the program (starters)

**Characteristic**	**Starters (Completed Week 1) (n = 8933)**	**Completers (Completed Week 6) (n = 1654)**	***P* value**
**Demographic Characteristic**	**No. (%)**	**No. (%)**	
** Gender**			0.04
Male	4302 (48)	770 (47)	
Female	4631 (52)	884 (53)	
			
** Marital Status**			< .001
Married or living with partner	5646 (63)	1125 (68)	
Single	3287 (37)	529 (32)	
			
** Family Status**			.002
With children	3808 (43)	646 (39)	
Without children	5125 (57)	1008 (61)	
			
** Age**	**Mean (SD)**	**Mean (SD)**	
Years	37.6 (9.8)	38.9 (9.6)	
			
**Clinical Characteristic**	**Mean (SD)**	**Mean (SD)**	
SADD^*^	12.31 (6.09)	11.52 (5.24)	< .001
Abbreviated APQ^†^	7.38 (5.01)	6.83 (4.66)	< .001
Core functioning	1.38 (0.82)	1.35 (0.74)	.17
Core problem	1.59 (0.92)	1.59 (0.81)	.93
Core well-being	1.62 (1.00)	1.57 (0.92)	.06
Core risk	0.41 (0.60)	0.34 (0.50)	< .001

^*^Short Alcohol Dependency Data Questionnaire

^†^Alcohol Problems Questionnaire

Of the program completers, 57% also fully completed the outcome questionnaires at the end of the 6-week program. The mean scores for the SADD and modified APQ were significantly lower for these people at week 6 than week 1, indicating that alcohol dependency and alcohol-related harm were significantly reduced at the end of the program (Table 4). Mental health symptoms were also significantly improved. At week 1, the mean item scores for the CORE-OM were at or above the clinical thresholds on all domains for both women and men, with the single exception of the risk score for men. At week 6, the mean item scores were significantly reduced across all scales, suggesting a reduction in mental health symptoms, a reduced risk of harm to self and others, and an improvement in subjective well-being and daily functioning (Table 4).

**Table 4:** Change in clinical outcomes between week 1 and week 6 in users who completed the 6-week program

**Clinical Outcome Measure**	**Week 1 (Baseline) Score**	**Week 6 (Final) Score**	***P* value**
	**Mean (SD)**	**Mean (SD)**	
**SADD (total score)**			
Men (n = 421)	11.51 (5.17)	7.65 (4.51)	< .001
Women (n = 520)	11.58 (5.35)	7.64 (5.04)	< .001
			
**APQ (total score)**			
Men (n = 421)	7.18 (4.74)	3.43 (3.90)	< .001
Women (n = 520)	6.61 (4.35)	3.05 (3.66)	< .001
			
**CORE-OM (item scores)**			
** Men (n= 421)**			
Functioning	1.32 (0.73)	0.87 (0.69)	< .001
Problem	1.51 (0.81)	0.94 (0.74)	< .001
Well-being	1.41 (0.90)	0.88 (0.84)	< .001
Risk	0.31 (0.46)	0.14 (0.33)	< .001
			
** Women (n = 520)**			
Functioning	1.37 (0.75)	0.85 (0.72)	< .001
Problem	1.66 (0.80)	0.99 (0.80)	< .001
Well-being	1.70 (0.91)	1.00 (0.89)	< .001
Risk	0.38 (0.55)	0.18 (0.44)	< .001

## Discussion

### Main Findings

This large pragmatic cohort study of users of a freely available online program for nondependent drinkers at risk of harm from alcohol use suggests that a small but significant (16.5% or 1 in 6) proportion of users will complete the 6-week program. Those that completed the program and provided outcome measures reported clinically significant benefit, with reduction in mean dependency and harm from alcohol, and improved mental health. Women, users with a partner, and users without children were more likely to complete the program.

### Relationship to Previous Literature

Our findings add to the growing body of literature suggesting that behavior change can be achieved through online interventions [34-36]. This suggestion provides some empirical support for the move toward providing online public health interventions [37]. In common with many other online interventions [38], we had a very high rate of attrition. As Eysenbach has pointed out, this may be integral to the nature of online interventions and does not undermine their potential usefulness, as the marginal cost per user is so low. We also found that the online intervention was frequently accessed outside normal working hours [39], although our findings on this point must be treated with caution, as a proportion of our users came from outside the United Kingdom, and the time of access was recorded using GMT.

This online service, in common with other studies [24,40], was used by as many women as men. This is an important difference from traditional alcohol treatment services, where women tend to be underrepresented and report that the services they receive do not meet their needs [41].

### Methodological Issues

This was a pragmatic cohort study aimed at exploring the usage patterns of a freely available online intervention. All data were self-reported, and we have no objective confirmation of any of the data reported here. Some users may have provided false or inaccurate data. However, there is no particular reason why registrants should have systematically lied at registration, and, given the large sample size (10000 registrants), the demographic data probably provide a reasonable description of the characteristics of users. Users who completed the 6-week course showed considerable motivation and commitment. The outcome measures were an integral part of the intervention, and users completed them entirely for their own benefit. It is difficult to know how truthful they were in completing these questionnaires, but there is some evidence to support the suggestion that responses to online questionnaires are comparable to traditional paper-based versions [42,43].

As this was an uncontrolled study, the data can only suggest an association between use of the program and an improvement in health outcomes. This study cannot determine whether this association was causal.

### Implications

If confirmed, these data suggest that an online intervention aimed at nondependent heavy drinkers can make a useful addition to the public health armamentarium. Although only 16% of those who registered completed the course, in public health terms this still represents a significant number of people who could benefit. The advantages of such an intervention are that the costs are unaffected by the number of users and that the intervention can be used at home, at any time of day or night, unlike more traditional services. Future work should explore whether adapting the program, including increasing the options for flexible use, removing the requirement to work through the elements sequentially, and introducing more personalized feedback, can improve “stickiness” or adherence to the program, and whether the association between use of the program and improved outcomes is causal. We are currently undertaking a randomized controlled trial to explore both these questions.

## Multimedia Appendix 1

Screenshots of the Down Your Drink program

## Multimedia Appendix 2

Recruitment leaflet sent to health care professionals

## Multimedia Appendix 3

Participant information sheet

## Abbreviations

APQ: Alcohol Problems Questionnaire
CORE-OM: Clinical Outcomes in Routine Evaluation-Outcome Measure
FAST: Fast Alcohol Screening Test
GMT: Greenwich mean time
SADD: Short Alcohol Dependency Questionnaire

## References

[1] Department of Health and Human Services (2000). 10th Special Report to the US Congress on Alcohol and Health.

[2] The Council of the European Union (2001). Council conclusions of 5 June 2001 on a community strategy to reduce alcohol-related harm. Off J European Communities.

[3] Cabinet Office Strategy Unit (2004). Alcohol Harm Reduction Strategy for England.

[4] Ballesteros Javier, Duffy John C, Querejeta Imanol, Ariño Julen, González-Pinto Asunción (2004). Efficacy of brief interventions for hazardous drinkers in primary care: systematic review and meta-analyses. Alcohol Clin Exp Res.

[5] Beich Anders, Thorsen Thorkil, Rollnick Stephen (2003). Screening in brief intervention trials targeting excessive drinkers in general practice: systematic review and meta-analysis. BMJ.

[6] Bien TH, Miller WR, Tonigan JS (1993). Brief interventions for alcohol problems: a review. Addiction.

[7] Heather Nick (2002). Effectiveness of brief interventions proved beyond reasonable doubt. Addiction.

[8] Moyer Anne, Finney John W, Swearingen Carolyn E, Vergun Pamela (2002). Brief interventions for alcohol problems: a meta-analytic review of controlled investigations in treatment-seeking and non-treatment-seeking populations. Addiction.

[9] Heather N (1996). The public health and brief interventions for excessive alcohol consumption: the British experience. Addict Behav.

[10] Beich Anders, Gannik Dorte, Malterud Kirsti (2002). Screening and brief intervention for excessive alcohol use: qualitative interview study of the experiences of general practitioners. BMJ.

[11] Ritterband L, Gonder-frederick LA, Cox DJ, Clifton AD, West RW, Borowitz S (2003). Internet interventions: in review, in use, and into the future. Prof Psychol Res Pract.

[12] Dutton WH, di Gennaro C, Hargrave AM (2005). The Oxford Internet Survey (OxIS) Report 2005: The Internet in Britain.

[13] Cunningham John A, Selby Peter L, Kypri Kypros, Humphreys Keith N (2006). Access to the Internet among drinkers, smokers and illicit drug users: is it a barrier to the provision of interventions on the World Wide Web?. Med Inform Internet Med.

[14] Hester R K, Delaney H D (1997). Behavioral Self-Control Program for Windows: results of a controlled clinical trial. J Consult Clin Psychol.

[15] Squires Daniel D, Hester Reid K (2004). Using technical innovations in clinical practice: the Drinker's Check-Up software program. J Clin Psychol.

[16] Rideout V (2001). Generation Rx.com: How Young People Use the Internet for Health Information.

[17] Cloud RN, Peacock PL (2001). Internet screening and interventions for problem drinking: results from the www.carebetter.com pilot study. Alcohol Treat Q.

[18] Cunningham J A, Humphreys K, Koski-Jännes A (2000). Providing personalized assessment feedback for problem drinking on the Internet: a pilot project. J Stud Alcohol.

[19] Saitz Richard, Helmuth Eric D, Aromaa Susan E, Guard Anara, Belanger Marc, Rosenbloom David L (2004). Web-based screening and brief intervention for the spectrum of alcohol problems. Prev Med.

[20] Hester Reid K, Miller Joseph H (2006). Computer-based tools for diagnosis and treatment of alcohol problems. Alcohol Res Health.

[21] Hester Reid K, Squires Daniel D, Delaney Harold D (2005). The Drinker's Check-up: 12-month outcomes of a controlled clinical trial of a stand-alone software program for problem drinkers. J Subst Abuse Treat.

[22] Kypri Kypros, Saunders John B, Williams Sheila M, Mcgee Rob O, Langley John D, Cashell-Smith Martine L, Gallagher Stephen J (2004). Web-based screening and brief intervention for hazardous drinking: a double-blind randomized controlled trial. Addiction.

[23] Cunningham John A, Humphreys Keith, Kypri Kypros, Van Mierlo Trevor (2006). Formative evaluation and three-month follow-up of an online personalized assessment feedback intervention for problem drinkers. J Med Internet Res.

[24] Koski-Jännes Anja, Cunningham John A, Tolonen Kari, Bothas Heikki (2007). Internet-based self-assessment of drinking--3-month follow-up data. Addict Behav.

[25] Linke S, Brown A, Wallace P (2004). Down your drink: a web-based intervention for people with excessive alcohol consumption. Alcohol Alcohol.

[26] Hodgson Ray J, John Bev, Abbasi Tina, Hodgson Rachel C, Waller Seta, Thom Betsy, Newcombe Robert G (2003). Fast screening for alcohol misuse. Addict Behav.

[27] Prochaska JO, Diclemente CC, Velicer WF, Ginpil S, Norcross JC (1985). Predicting change in smoking status for self-changers. Addict Behav.

[28] Dunn C, Deroo L, Rivara F P (2001). The use of brief interventions adapted from motivational interviewing across behavioral domains: a systematic review. Addiction.

[29] Monti P, Kadden R, Rohsenow D, Cooney N, Abrams D (2002). Treating Alcohol Dependence: A Coping Skills Training Guide.

[30] Marlatt GA, Gordon JR (1985). Relapse Prevention: Maintenance Strategies in the Treatment of Addictive Behaviors.

[31] Davidson R, Raistrick D (1986). The validity of the Short Alcohol Dependence Data (SADD) Questionnaire: a short self-report questionnaire for the assessment of alcohol dependence. Br J Addict.

[32] Williams B T, Drummond D C (1994). The Alcohol Problems Questionnaire: reliability and validity. Drug Alcohol Depend.

[33] Evans Chris, Connell Janice, Barkham Michael, Margison Frank, Mcgrath Graeme, Mellor-Clark John, Audin Kerry (2002). Towards a standardised brief outcome measure: psychometric properties and utility of the CORE-OM. Br J Psychiatry.

[34] Murray E, Burns J, See Tai S, Lai R, Nazareth I (2005). Interactive Health Communication Applications for people with chronic disease. Cochrane Database Syst Rev.

[35] Wantland Dean J, Portillo Carmen J, Holzemer William L, Slaughter Rob, Mcghee Eva M (2004). The effectiveness of Web-based vs. non-Web-based interventions: a meta-analysis of behavioral change outcomes. J Med Internet Res.

[36] Nguyen Huong Q, Carrieri-Kohlman Virginia, Rankin Sally H, Slaughter Robert, Stulbarg Michael S (2004). Internet-based patient education and support interventions: a review of evaluation studies and directions for future research. Comput Biol Med.

[37] Marshall A L, Owen N, Bauman A E (2004). Mediated approaches for influencing physical activity: update of the evidence on mass media, print, telephone and website delivery of interventions. J Sci Med Sport.

[38] Eysenbach Gunther (2005). The law of attrition. J Med Internet Res.

[39] Brennan P F, Moore S M, Smyth K A (1992). Alzheimer's disease caregivers' uses of a computer network. West J Nurs Res.

[40] Humphreys K, Klaw E (2001). Can targeting nondependent problem drinkers and providing internet-based services expand access to assistance for alcohol problems? A study of the moderation management self-help/mutual aid organization. J Stud Alcohol.

[41] Copeland J, Hall W (1992). A comparison of predictors of treatment drop-out of women seeking drug and alcohol treatment in a specialist women's and two traditional mixed-sex treatment services. Br J Addict.

[42] Ritter Philip, Lorig Kate, Laurent Diana, Matthews Katy (2004). Internet versus mailed questionnaires: a randomized comparison. J Med Internet Res.

[43] Miller Elizabeth T, Neal Dan J, Roberts Lisa J, Baer John S, Cressler Sally O, Metrik Jane, Marlatt G Alan (2002). Test-retest reliability of alcohol measures: is there a difference between internet-based assessment and traditional methods?. Psychol Addict Behav.

