# Supplementary material for: Internet-Based Interactive Health Intervention for the Promotion of Sensible Drinking: Patterns of Use and Potential Impact on Members of the General Public
Source: J Med Internet Res. 2007 May 8;9(2):e10. doi: 10.2196/jmir.9.2.e10 (PMC1874715; doi:10.2196/jmir.9.2.e10)
Supplement: Supplementary file 1 [file jmir_v9i2e10_app1.pdf]

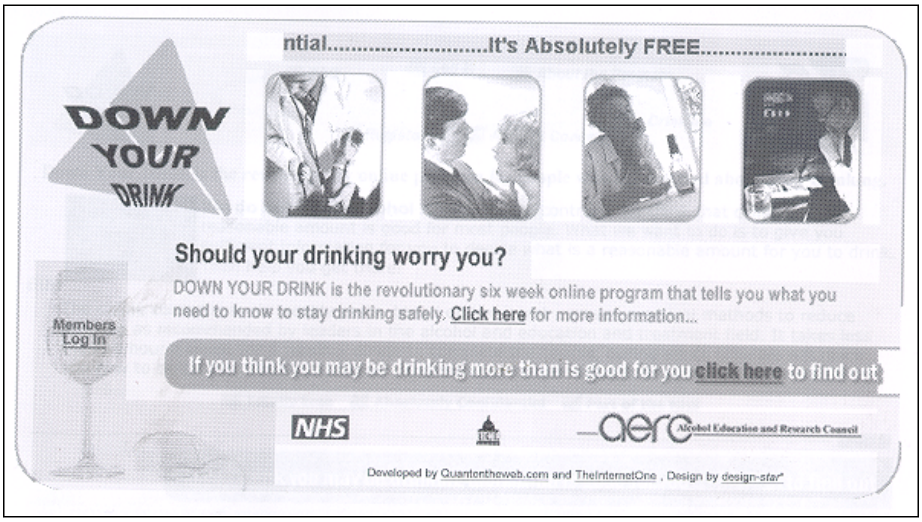
ntial..........................It's Absolutely FREE........................
DOWN YOUR DRINK
Should your drinking worry you?
DOWN YOUR DRINK is the revolutionary six week online program that tells you what you need to know to stay drinking safely. Click here for more information...
Members Log In
If you think you may be drinking more than is good for you click here to find out
NHS
UCL
aerc Alcohol Education and Research Council
Developed by Quantenthewob.com and TheInternetOne, Design by design-star

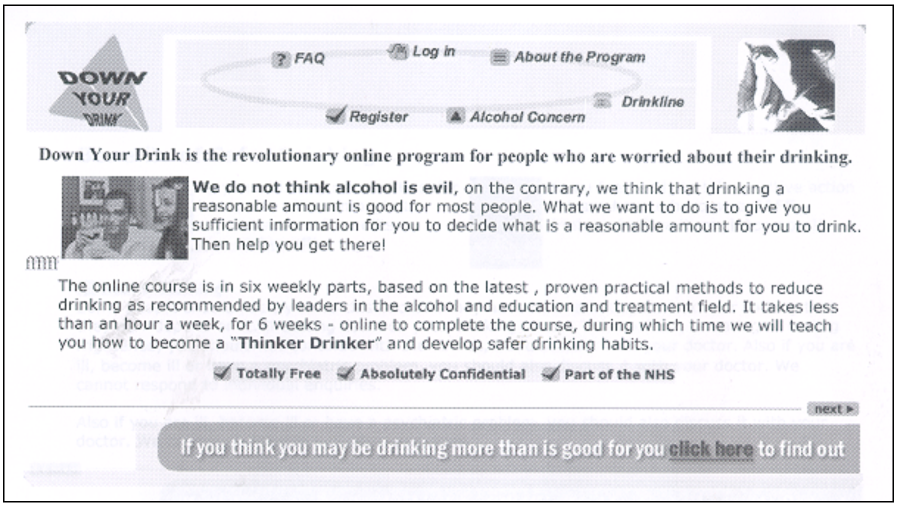
DOWN YOUR DRINK
FAQ
Log in
About the Program
Register
Alcohol Concern
Drinkline
Down Your Drink is the revolutionary online program for people who are worried about their drinking.
We do not think alcohol is evil, on the contrary, we think that drinking a reasonable amount is good for most people. What we want to do is to give you sufficient information for you to decide what is a reasonable amount for you to drink. Then help you get there!
The online course is in six weekly parts, based on the latest , proven practical methods to reduce drinking as recommended by leaders in the alcohol and education and treatment field. It takes less than an hour a week, for 6 weeks - online to complete the course, during which time we will teach you how to become a "Thinker Drinker" and develop safer drinking habits.
Totally Free
Absolutely Confidential
Part of the NHS
next
If you think you may be drinking more than is good for you click here to find out

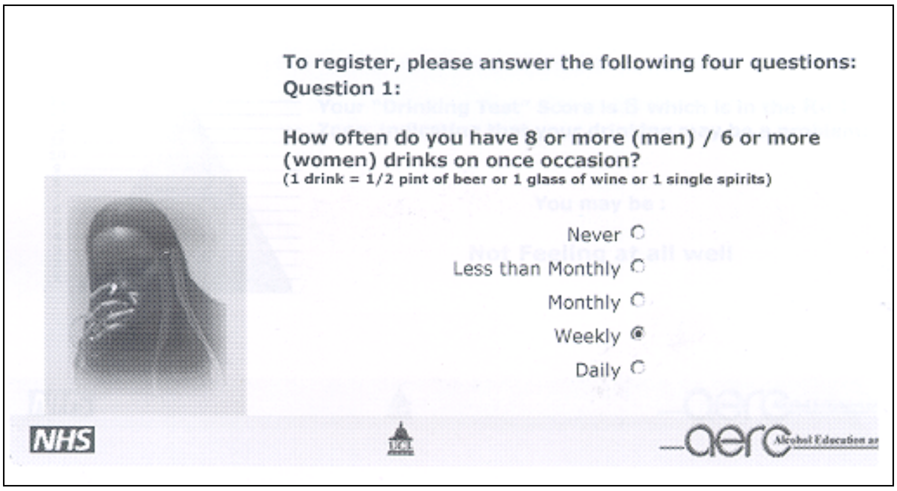
To register, please answer the following four questions:
Question 1:
How often do you have 8 or more (men) / 6 or more (women) drinks on once occasion?
(1 drink = 1/2 pint of beer or 1 glass of wine or 1 single spirits)
Never
Less than Monthly
Monthly
Weekly
Daily
NHS
UCL
aerc Alcohol Education a

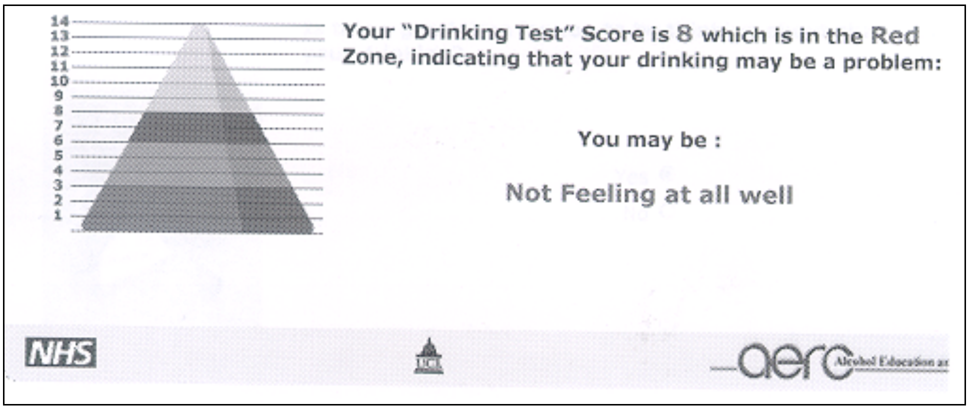
14
13
12
11
10
9
8
7
6
5
4
3
2
1
Your "Drinking Test" Score is 8 which is in the Red Zone, indicating that your drinking may be a problem:
You may be :
Not Feeling at all well
NHS
UCL
aerc Alcohol Education an

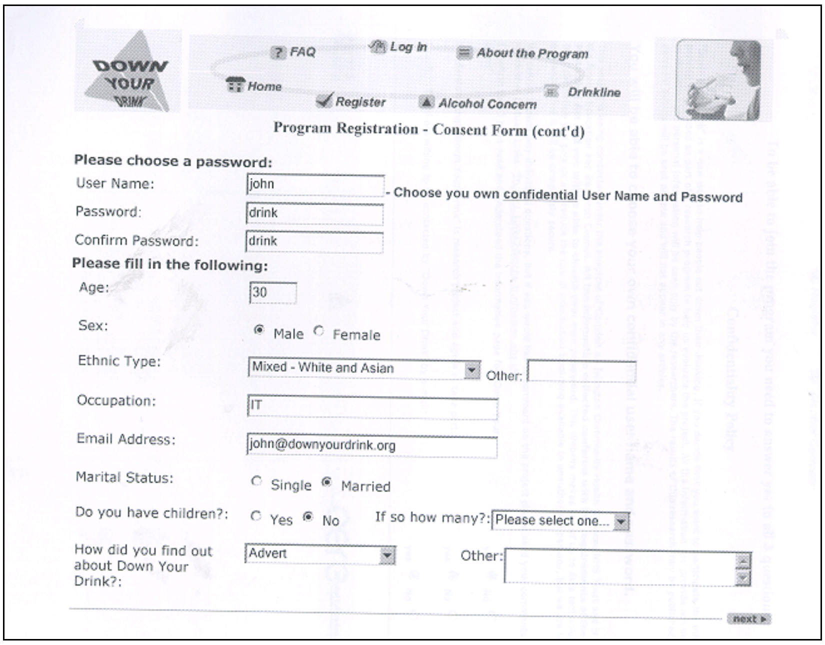
DOWN YOUR DRINK
FAQ
Log In
About the Program
Home
Register
Alcohol Concern
Drinkline
Program Registration - Consent Form (cont'd)
Please choose a password:
User Name: john - Choose you own confidential User Name and Password
Password: drink
Confirm Password: drink
Please fill in the following:
Age: 30
Sex: Male Female
Ethnic Type: Mixed - White and Asian Other:
Occupation: IT
Email Address: john@downyourdrink.org
Marital Status: Single Married
Do you have children?: Yes No If so how many?: Please select one...
How did you find out about Down Your Drink?: Advert Other:
next

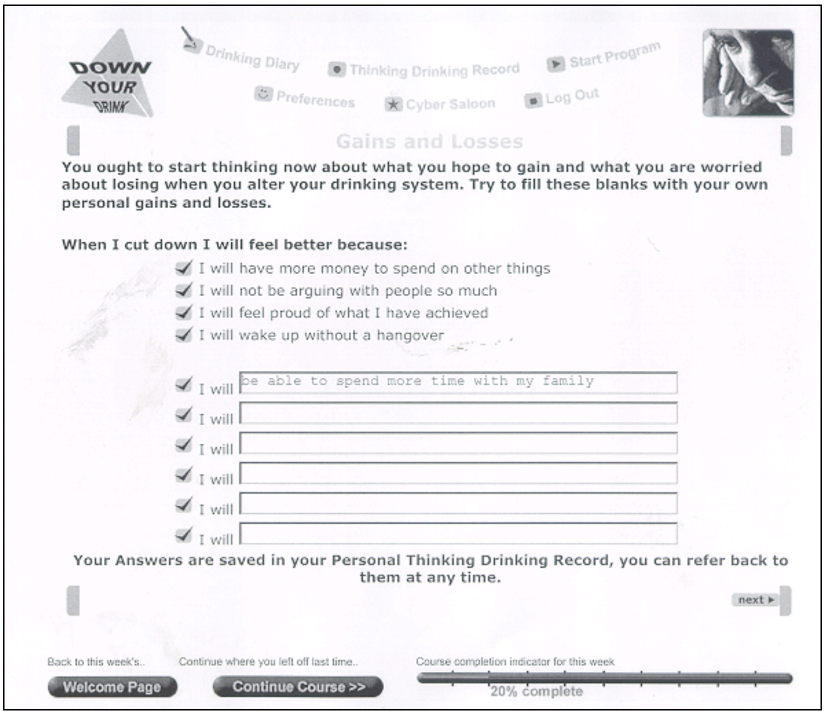
DOWN YOUR DRINK
Drinking Diary
Thinking Drinking Record
Start Program
Preferences
Cyber Saloon
Log Out
Gains and Losses
You ought to start thinking now about what you hope to gain and what you are worried about losing when you alter your drinking system. Try to fill these blanks with your own personal gains and losses.
When I cut down I will feel better because:
I will have more money to spend on other things
I will not be arguing with people so much
I will feel proud of what I have achieved
I will wake up without a hangover
I will be able to spend more time with my family
I will
I will
I will
I will
I will
Your Answers are saved in your Personal Thinking Drinking Record, you can refer back to them at any time.
next
Back to this week's..
Welcome Page
Continue where you left off last time..
Continue Course >>
Course completion indicator for this week
20% complete

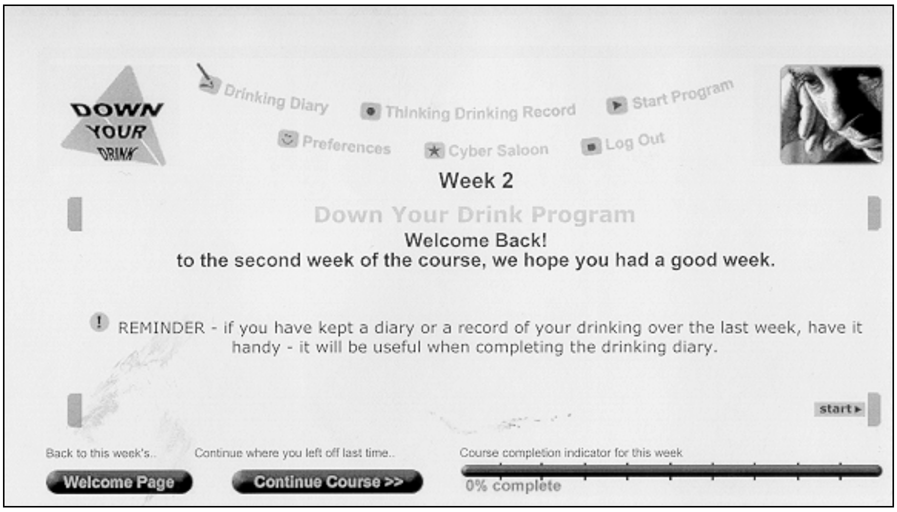
DOWN YOUR DRINK
Drinking Diary
Thinking Drinking Record
Start Program
Preferences
Cyber Saloon
Log Out
Week 2
Down Your Drink Program
Welcome Back!
to the second week of the course, we hope you had a good week.
REMINDER - if you have kept a diary or a record of your drinking over the last week, have it handy - it will be useful when completing the drinking diary.
start
Back to this week's..
Welcome Page
Continue where you left off last time..
Continue Course >>
Course completion indicator for this week
0% complete

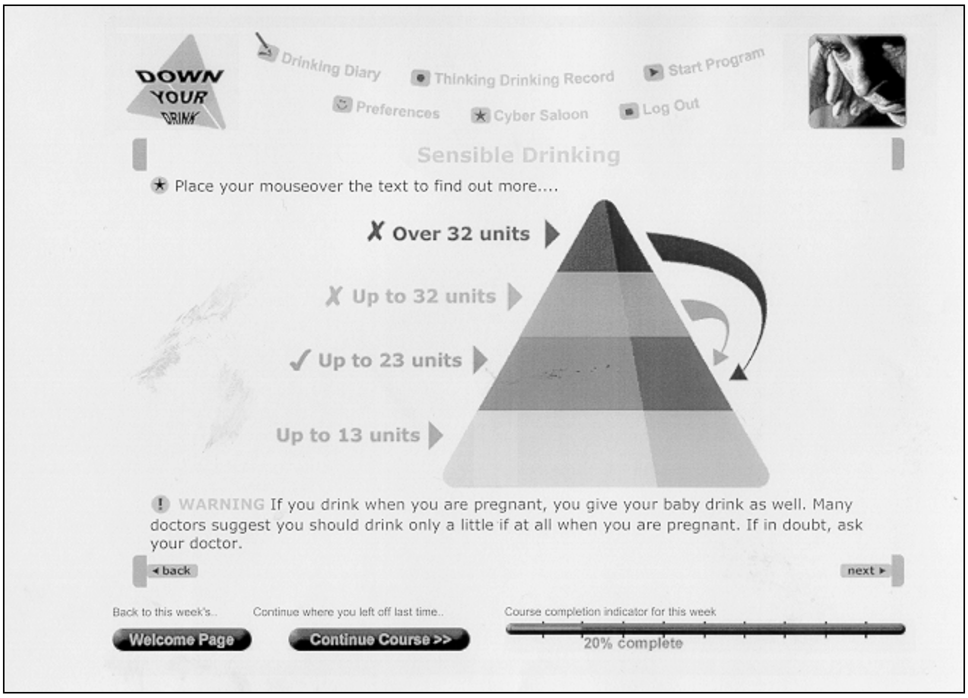
DOWN YOUR DRINK
Drinking Diary
Thinking Drinking Record
Start Program
Preferences
Cyber Saloon
Log Out
Sensible Drinking
Place your mouseover the text to find out more....
Over 32 units
Up to 32 units
Up to 23 units
Up to 13 units
WARNING If you drink when you are pregnant, you give your baby drink as well. Many doctors suggest you should drink only a little if at all when you are pregnant. If in doubt, ask your doctor.
back
next
Back to this week's..
Continue where you left off last time..
Course completion indicator for this week
Welcome Page
Continue Course >>
20% complete

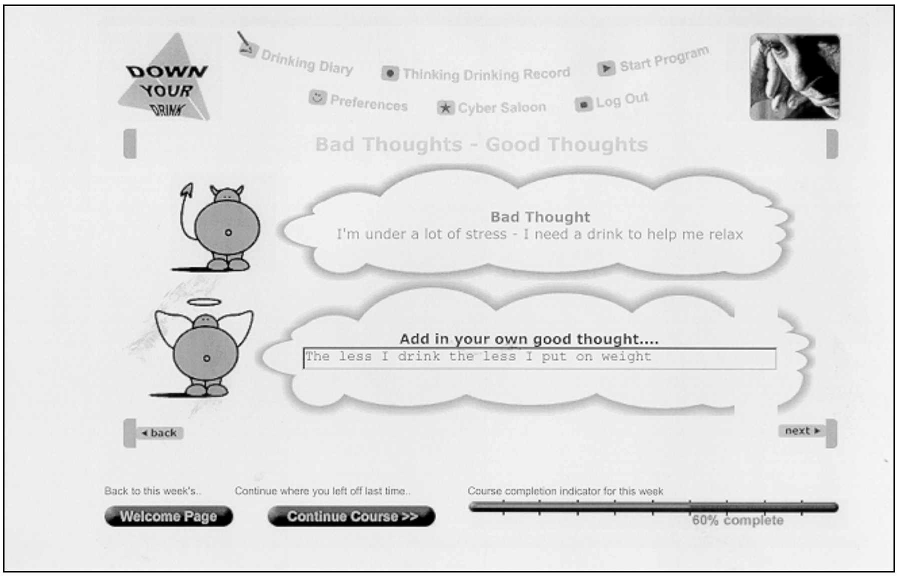
DOWN YOUR DRINK
Drinking Diary
Thinking Drinking Record
Start Program
Preferences
Cyber Saloon
Log Out
Bad Thoughts - Good Thoughts
Bad Thought
I'm under a lot of stress - I need a drink to help me relax
Add in your own good thought....
The less I drink the less I put on weight
back
next
Back to this week's..
Continue where you left off last time..
Course completion indicator for this week
Welcome Page
Continue Course >>
60% complete

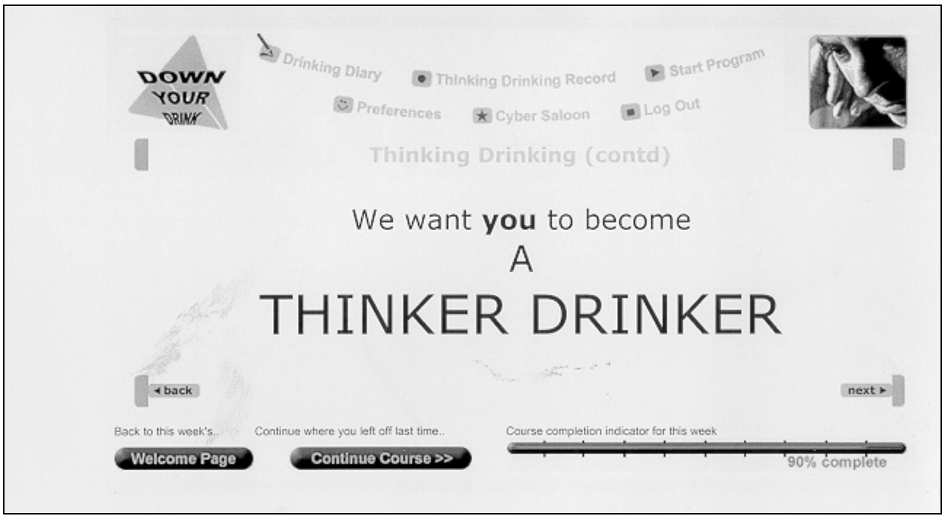
DOWN YOUR DRINK
Drinking Diary
Thinking Drinking Record
Start Program
Preferences
Cyber Saloon
Log Out
Thinking Drinking (contd)
We want you to become
A
THINKER DRINKER
back
next
Back to this week's..
Welcome Page
Continue where you left off last time..
Continue Course >>
Course completion indicator for this week
90% complete

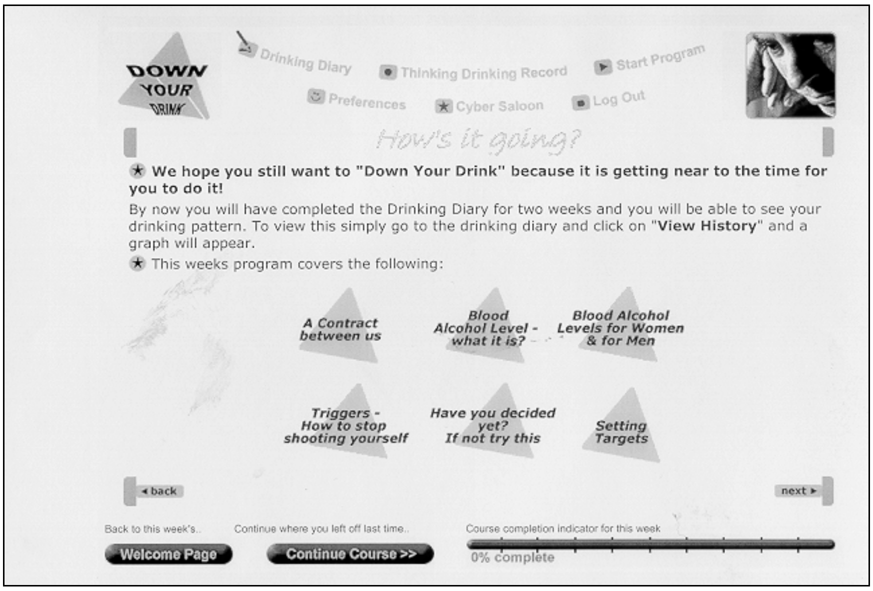
DOWN YOUR DRINK
Drinking Diary
Thinking Drinking Record
Start Program
Preferences
Cyber Saloon
Log Out
How's it going?
We hope you still want to "Down Your Drink" because it is getting near to the time for you to do it!
By now you will have completed the Drinking Diary for two weeks and you will be able to see your drinking pattern. To view this simply go to the drinking diary and click on "View History" and a graph will appear.
This weeks program covers the following:
A Contract between us
Blood Alcohol Level - what it is?
Blood Alcohol Levels for Women & for Men
Triggers - How to stop shooting yourself
Have you decided yet? If not try this
Setting Targets
back
next
Back to this week's..
Welcome Page
Continue where you left off last time..
Continue Course >>
Course completion indicator for this week
0% complete

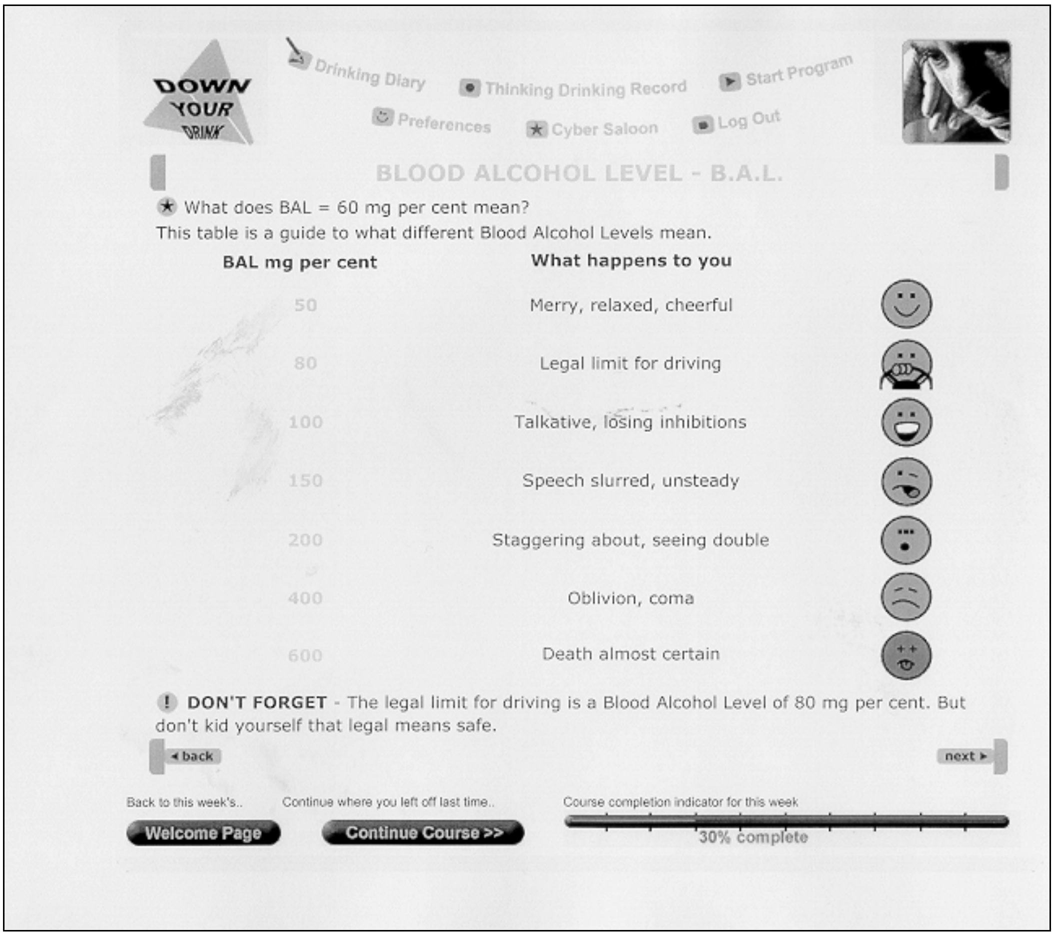

DOWN YOUR DRINK

Drinking Diary | Thinking Drinking Record | Start Program | Preferences | Cyber Saloon | Log Out

BLOOD ALCOHOL LEVEL - B.A.L.

What does BAL = 60 mg per cent mean?

This table is a guide to what different Blood Alcohol Levels mean.

BAL mg per cent	What happens to you
50	Merry, relaxed, cheerful
80	Legal limit for driving
100	Talkative, losing inhibitions
150	Speech slurred, unsteady
200	Staggering about, seeing double
400	Oblivion, coma
600	Death almost certain

DON'T FORGET - The legal limit for driving is a Blood Alcohol Level of 80 mg per cent. But don't kid yourself that legal means safe.

◂ back | next ▸

Back to this week's.. **Welcome Page** | Continue where you left off last time.. **Continue Course >>** | Course completion indicator for this week: 30% complete

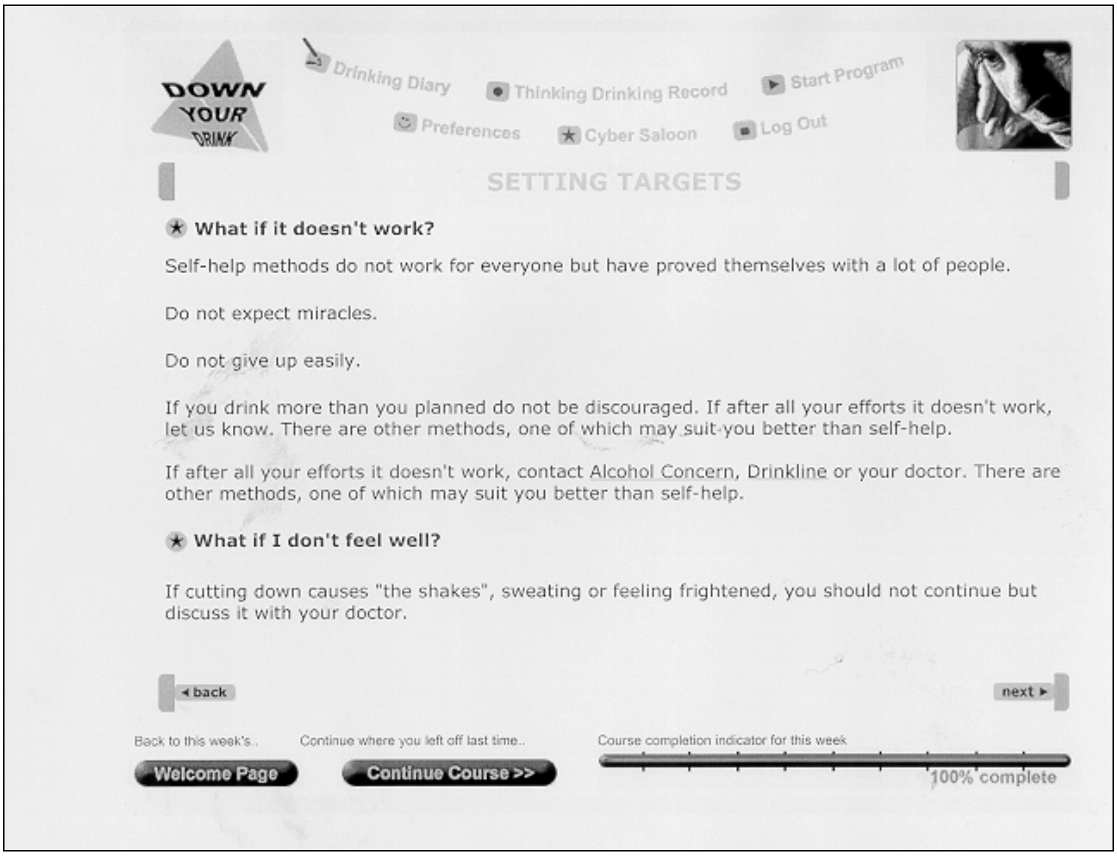

DOWN YOUR DRINK

Drinking Diary | Thinking Drinking Record | Start Program | Preferences | Cyber Saloon | Log Out

SETTING TARGETS

What if it doesn't work?

Self-help methods do not work for everyone but have proved themselves with a lot of people.

Do not expect miracles.

Do not give up easily.

If you drink more than you planned do not be discouraged. If after all your efforts it doesn't work, let us know. There are other methods, one of which may suit you better than self-help.

If after all your efforts it doesn't work, contact Alcohol Concern, Drinkline or your doctor. There are other methods, one of which may suit you better than self-help.

What if I don't feel well?

If cutting down causes "the shakes", sweating or feeling frightened, you should not continue but discuss it with your doctor.

◂ back | next ▸

Back to this week's.. **Welcome Page** | Continue where you left off last time.. **Continue Course >>** | Course completion indicator for this week: 100% complete

DOWN YOUR DRINK

Drinking Diary | Thinking Drinking Record | Start Program

Preferences | Cyber Saloon | Log Out

Rewards

★ **A lot of people find that it's very helpful to reward themselves for keeping to their target. If you're not sure about this, why not give it a try?**

Cutting down on your drinking brings its own rewards.

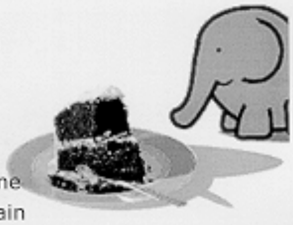

mmmm

- ★ ....like not waking up with a hangover
- ★ ....like not being tired and irritable all the time
- ★ ....like starting to enjoy talking to people again

★ **We think you deserve something EXTRA for all the effort you're making. So - if kept to your target for one day, you could -**

- ★ Watch a favourite video
- ★ Have a long soak in the bubble bath
- ★ Eat something you really like (if you're not slimming)
- ★ Phone up a friend
- ★ Listen to your favourite CD

★ **Enter your own daily rewards:**

- ★
- ★
- ★

◂ back

Back to this week's.. **Welcome Page**

Continue where you left off last time.. **Continue Course >>**

Course completion indicator for this week

40% complete
