# Supplementary material for: Internet-Based Interactive Health Intervention for the Promotion of Sensible Drinking: Patterns of Use and Potential Impact on Members of the General Public
Source: J Med Internet Res. 2007 May 8;9(2):e10. doi: 10.2196/jmir.9.2.e10 (PMC1874715; doi:10.2196/jmir.9.2.e10)
Supplement: Supplementary file 2 [file jmir_v9i2e10_app2.pdf]

The Revolutionary New Online Program to Help Problem Drinkers

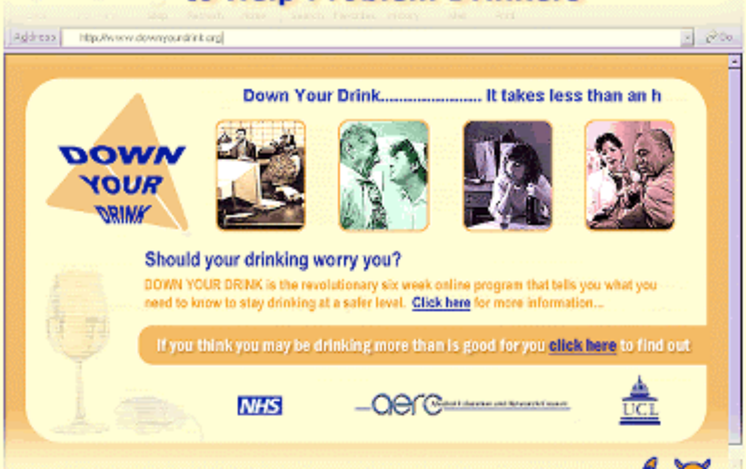

If you think you maybe drinking more than is good for you visit the website to find out.

★ The Down Your Drink self-help program is:

- *For anyone who is worried by their drinking and wishes to cut down*
- *Strictly confidential and free of charge*
- *In 6 weekly parts, taking less than an hour a week*
- *Accessible 24 hours a day from any PC connected to the Internet*
- *Based on the latest, proven practical methods to reduce drinking as recommended by leaders in the alcohol treatment and education field*

If you have patients who could benefit from learning how to become "Thinker Drinkers" pass on our web address:

www.downyour drink.org

To receive additional Down Your Drink leaflets
tel: 020 7530 2378 **e-mail:** info@downyourdrink.org

NHS aerc Alcohol Education and Research Council UCL

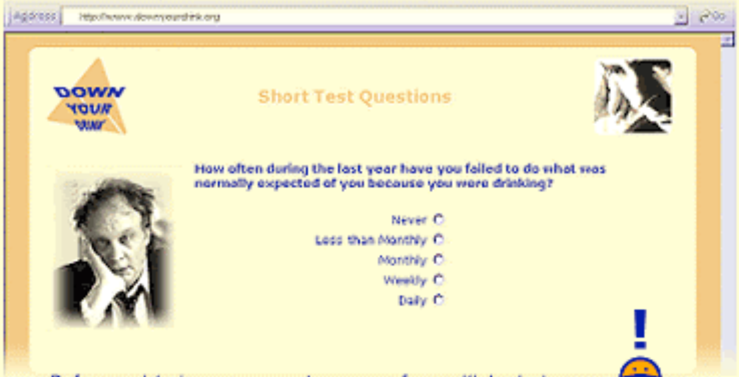

Before registering, users must answer a few multiple choice assessment questions. The site will immediately feed back personal drinking scores and "screen out" those for whom the course is not suitable.

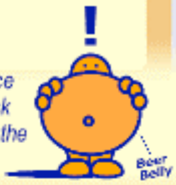

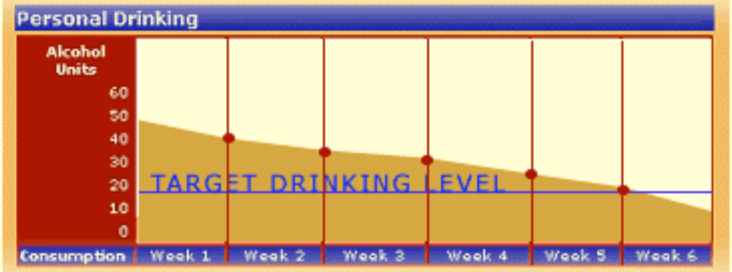

Down Your Drink members, decide their own drinking targets, keep a weekly drinking diary and decide when and where to complete the program.

If you know someone who could benefit from learning how to become "Thinker Drinkers" pass on our web address:

www.downyourdrink.org

To receive additional Down Your Drink leaflets

tel: 020 7530 2378 **e-mail:** info@downyourdrink.org

NHS — aerc Alcohol Education and Research Council — UCL
