# Supplementary material for: Internet-Based Interactive Health Intervention for the Promotion of Sensible Drinking: Patterns of Use and Potential Impact on Members of the General Public
Source: J Med Internet Res. 2007 May 8;9(2):e10. doi: 10.2196/jmir.9.2.e10 (PMC1874715; doi:10.2196/jmir.9.2.e10)
Supplement: Supplementary file 3 [file jmir_v9i2e10_app3.pdf]

PARTICIPANT INFORMATION PAGE (1)

"Down Your Drink" is a new way to help people cut down their drinking. If you decide that you want to participate the information you provide will be used as part of a research programme to help us evaluate the project. All the information you provide will be completely confidential and personal information will be seen only by the researchers. The results of this research may be published, but your personal details will be kept private and will not appear in any articles. We shall not pass your name or other details to anyone else.

This project is being conducted under the auspices of Camden & Islington Community Health Services NHS Trust and is supported by the Alcohol Education and Research Council. All the information collected conforms with the requirements of the Data Protection Act. This Website makes use of up to date technology to encrypt the information you give us and reduce the risk of information becoming available to unauthorised persons, but we are unable to guarantee that it will be completely secure

We cannot answer any individual questions but if you would like to comment on the project please send your comments to the lead researcher (Stuart Linke - S.Linke@………………).

You do not have to take part in this study if you do not want to. If you decide to take part you may withdraw at any time without having to give a reason.

CONSENT FORM (2)

"Down Your Drink"

1. I confirm that I have read and understood the information page for *"Down Your Drink"*

   Yes No (default No)
   ❍ ●

2. I confirm that ……..@………….. is my email address and that I can be contacted by *"Down Your Drink"*

   Yes No (default No)
   ❍ ●

3. I understand that *"Down Your Drink"* is a research project and agree to take part.

   Yes No (default No)
   ❍ ●

Camden and Islington Community Health Services NHS Trust

4, St Pancras Way, London, NW1 0PE
